# Arabidopsis SYT1 maintains stability of cortical endoplasmic reticulum networks and VAP27-1-enriched endoplasmic reticulum–plasma membrane contact sites

**DOI:** 10.1093/jxb/erw381

**Published:** 2016-10-17

**Authors:** Wei Siao, Pengwei Wang, Boris Voigt, Patrick J. Hussey, Frantisek Baluska

**Affiliations:** ^1^Institute of Cellular and Molecular Botany, University of Bonn, Kirschallee 1, D-53115 Bonn, Germany; ^2^Department of Biosciences, Durham University, South Road, Durham DH1 3LE, UK

**Keywords:** Cortical ER, cytoskeletons, ER stability, ER–PM contact sites, protein dynamics, synaptotagmins, VAP27.

## Abstract

Arabidopsis synaptotagmin 1 (SYT1) is localized on the endoplasmic reticulum–plasma membrane (ER–PM) contact sites in leaf and root cells. The ER–PM localization of Arabidopsis SYT1 resembles that of the extended synaptotagmins (E-SYTs) in animal cells. In mammals, E-SYTs have been shown to regulate calcium signaling, lipid transfer, and endocytosis. Arabidopsis SYT1 was reported to be essential for maintaining cell integrity and virus movement. This study provides detailed insight into the subcellular localization of SYT1 and VAP27-1, another ER–PM-tethering protein. SYT1 and VAP27-1 were shown to be localized on distinct ER–PM contact sites. The VAP27-1-enriched ER–PM contact sites (V-EPCSs) were always in contact with the SYT1-enriched ER–PM contact sites (S-EPCSs). The V-EPCSs still existed in the leaf epidermal cells of the *SYT1* null mutant; however, they were less stable than those in the wild type. The polygonal networks of cortical ER disassembled and the mobility of VAP27-1 protein on the ER–PM contact sites increased in leaf cells of the *SYT1* null mutant. These results suggest that SYT1 is responsible for stabilizing the ER network and V-EPCSs.

## Highlight

Arabidopsis synaptotagmin 1 is localized on ER–PM contact sites distinct from VAP27-1 and plays roles in maintaining ER morphology and the dynamics of VAP27-1.

## Introduction

In eukaryotic cells, proteins with multiple C2 domains are often found to participate in membrane trafficking or membrane-tethering processes, which can probably be Ca^2+^ regulated (Nalefski and Falke, 1996; Min *et al.*, 2007). Arabidopsis synaptotagmin 1 (SYT1) belongs to a five-member gene family (SYT1–SYT5), all of which contain an N-terminal transmembrane domain (TM), a synaptotagmin-like mitochondrial and lipid-binding protein (SMP) domain, and two tandem C2 domains at its C-terminus (Yamazaki *et al.*, 2010). The protein structure of Arabidopsis SYT1 is similar to that of both synaptotagmins (SYTs) and extended synaptotagmins (E-SYTs) in metazoans (Craxton, 2010).

At least 17 SYT isoforms have been found in mammals; most of them are expressed in neurons or neuroendocrine cells, and play essential roles in Ca^2+^-regulated neurotransmission and hormone secretion (Moghadam and Jackson, 2013). On the other hand, mammalian E-SYTs are endoplasmic reticulum (ER) membrane proteins that have similar functional domains to plant homologs (Min *et al.*, 2007). Human *E-SYT1* is expressed almost ubiquitously, while human *E-SYT2* and *E-SYT3* are expressed mainly in the cerebellum (Min *et al.*, 2007). Mammalian E-SYTs are known to participate in ER–plasma membrane (PM) tethering (Stefan *et al.*, 2013).

Several proteins localized on the ER–PM contact sites (EPCSs) in plants have been reported recently, including NETWORKED 3C (NET3C), VAMP/synaptobrevin-associated protein 27 (VAP27)-1, VAP27-3 and VAP27-4, and SYT1. NET3C belongs to the plant-specific NET superfamily of actin-binding proteins. All the 13 members of the Arabidopsis NET family contain a NET actin-binding (NAB) domain and various numbers of coiled-coil domains that can simultaneously interact with the actin filaments and different membrane compartments (Deeks *et al.*, 2012; Hawkins *et al.*, 2014; Wang *et al.*, 2014). Arabidopsis VAP27 proteins belong to the VAP33-like family which are homologs of mammalian VAPs and yeast suppressor of choline sensitivity (Scs2) (Sutter *et al.*, 2006; Saravanan *et al.*, 2009). The conserved major sperm (MSP) domain is essential for VAP27-1 to anchor on the ER–PM contact sites and for interaction of VAP27-1 with NET3C.

Arabidopsis SYT1 was thought to be a PM protein, but recent studies, as well as our results here, have shown that it is an ER-resident protein (Perez-Sancho *et al.*, 2015). Arabidopsis *SYT1* is constitutively expressed in all tissues, and the mutants are more sensitive to salt, freezing, and mechanical stresses (Schapire *et al.*, 2008; Yamazaki *et al.*, 2008, 2010; Levy *et al.*, 2015; Perez-Sancho *et al.*, 2015). In addition, virus infections are delayed in *SYT1* null mutants (Lewis and Lazarowitz, 2010; Uchiyama *et al.*, 2014). Arabidopsis SYT1 and VAP27-1 have also been shown to interact with the plasmodesmata-resident reticulons (Kriechbaumer *et al.*, 2015). However, the relationship between Arabidopsis SYT1 and VAP27-1 on the ER–PM contact sites is still unclear.

This work addresses the spatial relationships between Arabidopsis SYT1, VAP27-1, and microtubules on the cell cortex. By live-cell imaging, immunocytochemistry, and immunogold labeling, SYT1 and VAP27-1 are shown to be localized on distinct ER–PM contact sites. V-EPCSs are always in contact with S-EPCSs and often associated with microtubules, but S-EPCSs are often excluded by microtubules on the cell cortex and often arranged along thick actin filaments. Amino acid substitutions demonstrate that VAP27-1 mutant protein has no dominant-negative effect on the SYT1 anchoring to the PM. Using stable transformation of VAP27-1 in Arabidopsis, SYT1 is shown to be essential for maintaining the stability of the ER network and the ER–PM contact sites. The dynamics of VAP27-1 on the ER–PM contact sites are restrained by SYT1. In summary, this study shows that Arabidopsis SYT1 is critical for tethering the ER to the PM and plays roles in regulating the ER remodeling and the stability of V-EPCSs.

## Materials and methods

### Plant material and growth conditions

*Arabidopsis thaliana* (L.) seedlings were grown on vertical half-strength Murashige and Skoog (1/2 MS) agar plates (pH 5.8) in a growth chamber at 22 °C under long-day conditions (16 h light/8 h dark). After 14 d, the seedlings were transferred and grown in pots in a culture room at 22 °C under long-day conditions (16 h light/8 h dark). Experiments were performed using *A. thaliana* Columbia ecotype (Col-0), *syt1-2* (SAIL_775_A08), the *SYT1 promoter:SYT1-GFP/Col-0* transgenic line (Yamazaki *et al.*, 2010), *VAP27-1* RNAi knock-down lines, and *VAP27-1-YFP*/Col-0 transgenic lines (Wang *et al.*, 2014, 2016). *VAP27-1-YFP*/*syt1-2* transgenic lines were obtained by *Agrobacterium*-mediated transformation of *VAP27-1-YFP* into *syt1-2* using floral dipping (Clough and Bent, 1998; Zhang *et al.*, 2006). For FM4-64 staining and brefeldin A (BFA) treatment, roots of 4-day-old Arabidopsis seedlings were transferred into 1/10 MS solution and pre-cooled at 6 °C for 5 min. After staining with 4.1 µM FM4-64 (SynaptoRed™ C2, Sigma) at 6 °C for 10 min, the roots were incubated with 35.6 µM BFA at room temperature for 60 min. *Z*-stack images of the roots at 2 µm intervals were acquired by confocal microscopy.

### Constructs

Binary plasmids of Arabidopsis SYT1 tagged with green fluorescent protein (GFP) driven by the native *SYT1* promoter (SYT1–GFP), VAP27-1 tagged with yellow fluorescent protein (YFP) driven by the 35S promoter (VAP27-1–YFP), and NET3C fused with red fluorescent protein (RFP) driven by the 35S promoter (RFP–NET3C) were described previously (Yamazaki *et al.*, 2010; Wang *et al.*, 2014). The ER marker RFP–HDEL and cyan fluorescent protein (CFP)–HDEL (Lee *et al.*, 2013; Wang *et al.*, 2014), the Golgi marker sialyltransferase (ST)–RFP (Renna *et al.*, 2005; Schoberer *et al.*, 2010), the early endosome marker CLC–mCherry (Wang *et al.*, 2013; Wang *et al.*, 2015), the microtubule marker MBD-MAP4 (microtubule-binding domain of microtubule-associated protein 4)–DsRed (Marc *et al.*, 1998; Granger and Cyr, 2001), and the actin maker ABD2–mCherry (Voigt *et al.*, 2005) were described in the indicated reports.

### Agrobacterium-*mediated transient expression in tobacco leaves*

*Nicotiana benthamiana* plants were grown in a culture room at 22 °C under long-day conditions (16 h light/8 h dark) for 3–4 weeks. Each construct was transformed into *Agrobacterium tumefaciens* strain GV3101::pMP90 by electroporation followed by selection on YEB plates containing the appropriate antibiotics. A single colony was inoculated and grown overnight in 3 ml of YEB liquid medium with antibiotics at 37 °C. A 1 ml aliquot of bacterial culture was centrifuged at 3500 rpm for 5 min and the pellet was resuspended in 1 ml of infiltration medium (20 mM citric acid, 2% sucrose, and 0.2 mM acetosyringone). The bacterial suspension was centrifuged and the pellet was resuspended again in 1 ml of infiltration medium to ensure complete removal of remnant antibiotics. Absorbance of the suspension at 600 nm was measured and the OD_600_ was adjusted to the specified value for infiltration (OD_600_=0.2 for SYT1–GFP, CLC–mCherry, MAP4–DsRed, and ABD2–mCherry; OD_600_=0.1 for VAP27-1–YFP, NET3C–RFP, ST–RFP, RFP–HDEL, and CFP–HDEL). Syringe infiltration of tobacco leaves was performed as previously described (Batoko *et al.*, 2000; Sparkes *et al.*, 2006). The plants were kept in the same culture room after infiltration for 2 d before confocal imaging. The PM staining was conducted by infiltrating 4.1 µM FM4-64 (SynaptoRed™ C2, Sigma) in Milli-Q water into the leaves 5 min prior to microscopy.

### FRAP analysis

Stable transgenic Arabidopsis expressing VAP27-1–YFP in the Col-0 (*VAP27-1-YFP*/Col-0) and *syt1-2* (*VAP27-1-YFP*/*syt1-2*) background were grown in pots for 4 weeks. One T_3_ homozygous line of VAP27-1/Col-0 and five T_1_ heterozygous lines of VAP27-1/*syt1-2* were planted. The expression levels of VAP27-1–YFP in five VAP27-1/*syt1-2* heterozygous lines were examined by confocal microscopy, and one with comparable expression of VAP27-1–YFP with that in VAP27-1/Col-0 was used for FRAP (fluorescence recovery after photobleaching) experiments. Leaf discs (0.5 × 0.5 cm^2^) from the first or second leaf of the 4-week-old Arabidopsis were selected because the leaves have a flattened surface. The leaf discs were mounted in Milli-Q water and analyzed using a confocal microscope with a ×60 oil immersion objective and a zoom factor of 5.0. Confocal parameters were identical for all the FRAP experiments. Two percent transmission of an argon laser at 515 nm was used for imaging and 80% transmission for photobleaching. Ten reference scans were taken before bleaching and 60 scans were taken after bleaching at 3 s intervals. At least 20 VAP27-1–YFP-labeled puncta in each line were analyzed. The raw data were normalized and the best-fit curves were generated by least-squares regression using Prism (Graumann *et al.*, 2007; Wang *et al.*, 2011).

### Western blot

Fourteen-day-old seedlings were frozen by liquid nitrogen and ground into powder. The total protein was extracted with protein extraction buffer [50 mM Tris–HCl, 150 mM NaCl, 10 mM MgCl_2_, 0.5% NP-40, 1 mM phenylmethylsulphonyl fluoride (PMSF), and 1× protease inhibitor cocktail (P9599, Sigma)]. The protein samples were quantified using Bio-Rad Bradford Protein Assay and subjected to 5× sample buffer (300 mM Tris–HCl, 60% glycerol, 10% SDS, 500 mM DTT, and 0.01% bromphenol blue). The protein was denatured by heating at 70 °C for 5 min and cooled down on ice. A 40 µg aliquot of protein was loaded to gels for SDS–PAGE analysis and transferred onto a PVDF (polyvinylidene difluoride) membrane by electroblotting. The membranes were first stained with Ponceau S, imaged, and then blocked with 4% non-fat milk+4% BSA in Tris-buffered saline with Tween-20 (TBST; 10 mM Tris, 150 mM NaCl, and 0.1% Tween-20, pH 7.6) for 60 min. After incubation with antibodies against SYT1 (1:1000) or VAP27-1 (1:1500) at 4 °C for 16 h, the membranes were washed three times for 10 min and incubated with horseradish peroxidase (HRP)-conjugated anti-rabbit or HRP-conjugated anti-mouse antibodies at room temperature for 1.5 h. The blots were washed three times with TBST for 10 min, and the proteins were visualized using an ECL imaging system (LAS-1 000, Fuji Films). SYT1 antibody was kindly provided by Professor Miguel A. Botella, Universidad de Malaga, Spain (Perez-Sancho *et al.*, 2015), and VAP27-1 antiserum was described previously (Wang *et al.*, 2014, 2016).

### Immunogold labeling

Root tips of Arabidopsis, with or without pre-treatment with 50 µM BFA for 2 h, were fixed using a high-pressure freezing machine (Bal-Tec HPM010, Balzers, Liechtenstein), freeze-substituted at −80 °C, and embedded in Lowicryl^®^ Embedding Media HM20 (Polysciences, Warrington, PA, USA). After blocking and incubation with antibodies against SYT1 (1:150) and VAP27-1 (1:150) overnight at 4 °C, the ultrathin sections were rinsed and incubated with 15 nm gold particle-conjugated anti-rabbit and 6 nm gold particle-conjugated anti-mouse antibodies at room temperature for 2 h. The sections were extensively washed and stained with uranyl acetate. The samples were imaged with an LEO 912AB electron microscope (ZEISS AG, Oberkochen). For statistical analysis, the positive gold signals were counted along the PM (within a distance of 50 nm apart from the PM). A region of interest (ROI) was defined as a rectangular area with a width of 50 nm (apart from the PM) and a length of 100 nm along the PM. More than one positive signal within a ROI was defined as a clustered labeling (co-localization).

### Whole-mount immunofluorescence labeling

Five-day-old Arabidopsis seedlings were fixed in fixation buffer (1.5% paraformaldehyde+0.5% glutaraldehyde in 1/2 microtubule-stabilizing buffer (MTSB; 50 mM PIPES, 5 mM MgSO_4_, and 5 mM EGTA, pH 6.9) with vacuum filtration for 1 h, and the fixed seedlings were washed once with 1/2 MTSB and twice with phosphate-buffered saline (PBS; 140 mM NaCl, 2.7 mM KCl, 6.5 mM Na_2_HPO_4_, and 1.5 mM KH_2_PO_4_, pH 7.3) for 10 min. After three reductions with sodium borohydride (NaBH_4_) in PBS, the roots were washed three times for 5 min and then incubated with 2% driselase+2% cellulose+1% pectolyase in PBS at 37 °C for 30 min. The cells were permeabilized by incubating with 10 mM glycine three times for 5 min and 2% NP-40+10% DMSO for 1 h in PBS. The roots were washed with PBS for 10 min and then blocked with 2% BSA in PBS. After incubation with antibodies against SYT1 (1:200) and VAP27-1 (1:200) at 4 °C for 16 h, the roots were washed six times for 10 min and incubated with Cy5^®^-conjugated anti-rabbit and Alexa Fluor^®^ 488-conjugated anti-mouse antibodies at 37 °C for 1.5 h then at room temperature for 1.5 h. For single SYT1 immunolabeling, Alexa Fluor^®^ 488-conjugated anti-rabbit antibody was used. The roots were washed six times for 10 min, and the nuclei were stained with 5 µM DAPI in PBS. The roots were washed twice with PBS for 5 min before confocal imaging.

### Confocal microscopy

Confocal imaging was performed by using an Olympus FluoView™ FV1000 confocal microscope equipped with diode (405 nm), argon ion (458, 488 and 514 nm), and helium–neon (543 nm) lasers. A GFP single image was excited at 488 nm and the emission signals were collected from 500 nm to 600 nm. YFP was excited at 515 nm and emission was collected from 530 nm to 630 nm. The red fluorescent dye FM4-64 was excited at 543 nm and emission was filtered between 660 nm and 760 nm. For simultaneous imaging of CFP, GFP, and FM4-6, the setting of CFP (Ex 405 nm/Em 440–505 nm), GFP (Ex 488 nm/Em 510–550 nm), and FM4-64 (Ex 543 nm/Em 600–660 nm) was used. For simultaneous imaging of GFP, YFP, and mCherry, the setting of GFP (Ex 458 nm/Em 470–515 nm), YFP (Ex 514 nm/Em 530–580 nm), and mCherry (Ex 543 nm/Em 600–660 nm) was used.

### Accession numbers

Arabidopsis *SYT1* (AT2G20990); *VAP27-1* (AT3G60600); *NET3C* (AT2G47920)

## Results

### Arabidopsis SYT1 is localized on the ER–PM contact sites

**Fig. 1.**
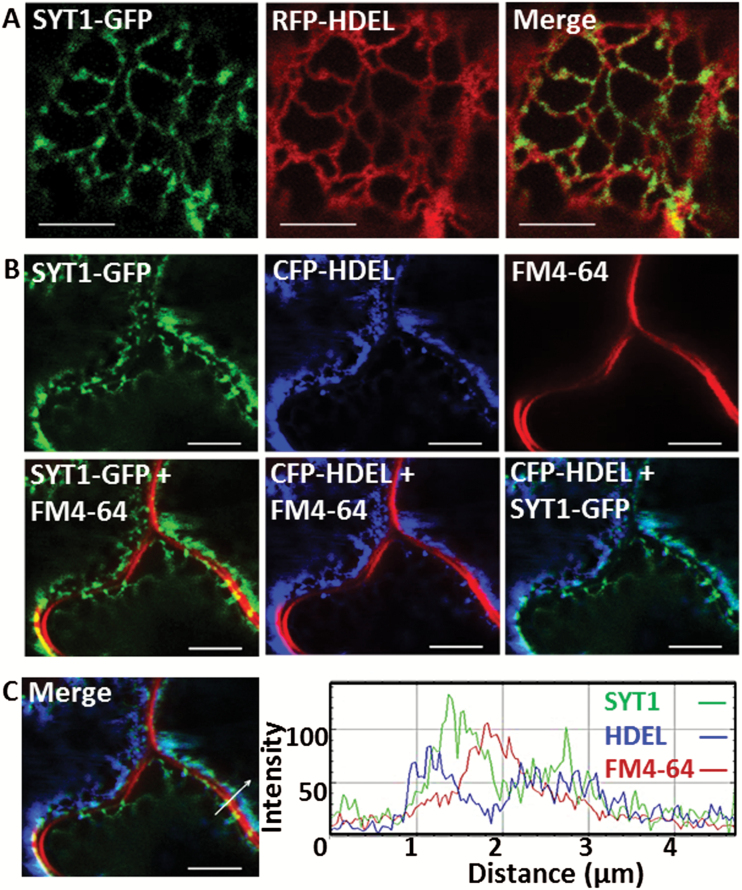
SYT1 unevenly distributes on the cortical ER and forms stable attachments to the PM in *N. benthamiana* leaf epidermis. (A) Co-expression of SYT1–GFP and RFP–HDEL shows that the stable SYT1 puncta are localized on ER tubules and cisternae. (B) ER-resident SYT1 attaches to the FM4-64-stained PM at the immotile ER–PM contact sites. (C) The intensity profiles of the cells in (B) show that SYT1 signal peaks between the ER lumen marker HDEL and the PM marker FM4-64 at the ER–PM contact sites. Scale bars=5 µm.

Arabidopsis SYT1 and VAP27-1 have been shown to be ER–PM-tethering proteins. However, the relationship between SYT1 and VAP27-1 remains unclear. To gain a better understanding of this relationship, SYT1–GFP was first transiently co-expressed with the ER lumen markers RFP–HDEL or CFP–HDEL in leaves of *N. benthamiana*. Most SYT1–GFP signals were found to accumulate on stable spots along the relatively stationary ER tubules and cisternae, while a lower amount of SYT1–GFP was detected on the motile, quickly remodeling ER strands (Fig. 1A; Supplementary Movie S1 at *JXB* online). The co-expression of CFP–HDEL followed by FM4-64 staining showed that SYT1–GFP was localized on the ER and attached to the PM at specific stationary regions, namely the ER–PM contact sites (Fig. 1B, C). This observation is in agreement with previous studies (Levy *et al.*, 2015; Perez Sancho *et al.*, 2015) and immunogold labeling of endogenous proteins (discussed later).

Studies have shown that Arabidopsis SYT1 plays roles in regulating the endocytosis in the leaves of *N. benthamiana* (Lewis and Lazarowitz, 2010) and the secretory pathway in Arabidopsis (Kim *et al.*, 2016). However, the functions of SYT1 on the endocytic pathway in Arabidopsis remain unclear. To investigate whether SYT1 would be incorporated in the endocytic vesicles, SYT1–GFP was co-expressed with the Golgi marker (ST–RFP) and the early endosome marker (CLC–mCherry). The results showed that SYT1 was not translocated into the Golgi apparatus and the clathrin-coated vesicles (Supplementary Fig. S1A, B). In addition, the roots of transgenic Arabidopsis expressing SYT1–GFP driven by the *SYT1* native promoter and VAP27-1–GFP driven by the 35S promoter were stained with the lipophilic styryl dye FM 4-64 followed by BFA treatment. BFA is a fungal toxin that blocks the secretory vesicle trafficking through the ER and the Golgi apparatus. The blockage will lead to the accumulation of *trans*-Golgi networks/early endosomes (TGNs/EEs) and the Golgi apparatus and form the BFA compartments (Berson *et al.*, 2014; Naramoto *et al.*, 2014). The results showed that neither SYT1–GFP nor VAP27-1–GFP was co-localized with the BFA compartments (Supplementary Fig. S1C, D), indicating that these two proteins were not incorporated into the endosomes. Cryo-immunogold electron microscopy further confirmed that both SYT1 and VAP27-1 were still localized on the ER–PM contact sites in the BFA-treated root cells (Supplementary Fig. S1E, F).

### SYT1 and VAP27-1 are localized on different regions of ER–PM contact sites

**Fig. 2.**
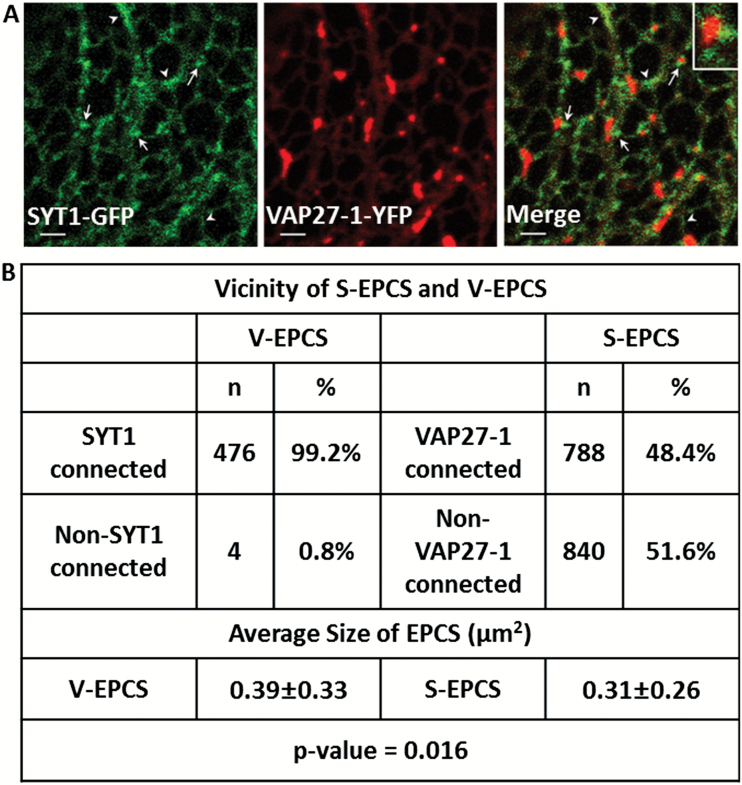
SYT1 and VAP27-1 are localized on different regions of ER–PM contact sites in *N. benthamiana* leaf epidermis. (A) The VAP27-1-enriched ER–PM contact sites (V-EPCSs) do not overlap with the SYT1-enriched ER–PM contact sites (S-EPCSs) (arrows). SYT1 partly overlaps with VAP27-1 on the motile ER tubules or strands (arrowheads). The inset shows a V-EPCS surrounded by the S-EPCSs. Scale bar=2 µm. (B) Spatial relationship between S-EPCSs and V-EPCSs. Almost all V-EPCSs are in contact with S-EPCSs, but about half of S-EPCSs are localized on the ER without connection with V-EPCSs. The average size of V-EPCS is slightly larger than that of S-EPCS; however, the sizes of EPCSs vary wildly from cell to cell, as indicated by the high standard deviations (±SD). Sixteen cells from three independent experiments are analyzed.

To examine whether SYT1 and VAP27-1 are localized on the same ER–PM contact sites, SYT1–GFP and VAP27-1–YFP were transiently co-expressed in leaves of *N. benthamiana*. The result showed that the signal of SYT1–GFP did not completely overlap with that of VAP27-1–YFP at EPCSs; however, it was often found localized around VAP27-1–YFP-labeled EPCSs (Fig. 2A, arrows; Supplementary Movie S2). On the other hand, SYT1–GFP was mainly overlapped with VAP27-1–YFP on the ER strands, tubules, or cisternae, but not on ER–PM contact sites (Fig. 2A, arrowheads; Supplementary Movie S2). Statistical analyses showed that 99.17% (476 out of 480) of VAP27-1 puncta were found to be associated with SYT1 puncta, whereas only 48.40% (788 out of 1628) of SYT1 puncta were in contact with VAP27-1 puncta (Fig. 2B). It has been shown that overexpression of VAP27-1 and NET3C together in *N. benthamiana* causes the enlargement of the V-EPCSs (Wang *et al.*, 2016). To illustrate further the relationships between SYT1, VAP27-1, and NET3C, co-expression of these proteins in tobacco leaves was determined. Co-expression of SYT1–GFP, VAP27-1–YFP, and RFP–NET3C showed that VAP27-1–YFP and NET3C–RFP were co-localized on the same ER–PM contact sites, and SYT1–GFP was found closely surrounding it; little co-localization was observed (Supplementary Fig. S2). Please note that VAP27-1 and NET3C interaction enlarges the size of EPCSs as described in Wang *et al.* (2016). Previous studies have shown that VAP27-1 interacts with microtubules (Wang *et al.*, 2014) while SYT1 is often excluded by microtubules (Perez Sancho *et al.*, 2015). In order to illustrate the relationships between SYT1, VAP27-1, and the cytoskeleton, co-expression of these proteins and the cytoskeletal markers in *N. benthamiana* leaves was examined. The results showed that VAP27-1–YFP puncta were often associated with the MBD-MAP4–DsRed-labeled microtubules and in close proximity to the microtubule-depleted region-located SYT1 puncta (Supplementary Fig. S3A). SYT1–GFP puncta are often arranged along the ABD2–mCherry-labeled F-actin (Supplementary Fig. S3B). A VAP27-1 punctum sandwiched by two SYT1 puncta and penetrated by one microtubule is shown in Supplementary Fig. S3A. The aforementioned data indicated that SYT1 might play a role in controlling the formation, the size, or the stability of VAP27-1-located EPCSs.

**Fig. 3.**
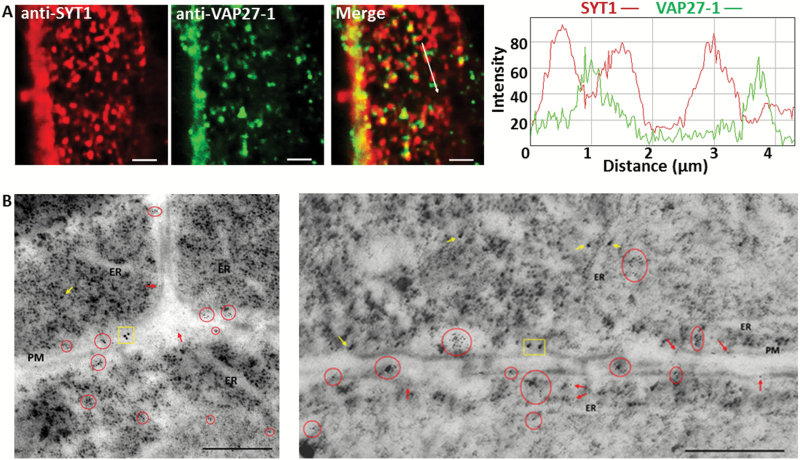
SYT1 and VAP27-1 accumulate on different regions of the ER–PM contact sites in Arabidopsis root cells. (A) Whole-mount immunocytochemistry of the root cells in wild-type Arabidopsis shows that SYT1 and VAP27-1 puncta are in close proxity on the cell cortex. The intensity profiles show that the peaks of SYT1 and VAP27-1 signals are shifted. Scale bars=2 µm. (B) Double immunogold labeling of SYT1 and VAP27-1 in Arabidopsis roots. The electron micrographs of ultrathin cryosections of the wild-type Arabidopsis root cells and immunogold labeling show that SYT1 (15 nm gold particles) and VAP27-1 (6 nm gold particles) are not localized on the same regions along the PM. The clusters of gold particles are highlighted as SYT1-clusters (yellow rectangles) and VAP27-1-clusters (red circles). SYT1 single labeling (yellow arrows) and VAP27-1 single labeling (red arrows) are indicated. Scale bars=500 nm.

To obtain more convincing evidence in *A. thaliana*, whole-mount immunofluorescent labeling using SYT1- and VAP27-1-specific antibodies (Wang *et al.*, 2014; Perez-Sancho *et al.*, 2015) was conducted to probe the native SYT1 and VAP27-1 proteins in the roots of wild-type Arabidopsis. The images showed that SYT1 and VAP27-1 were localized on different regions of the cortical ER (Fig. 3A). Immunofluorescent staining of the root cells in wild-type Arabidopsis with SYT1-specific antibody showed clear puncta signals on the cell cortex (Supplementary Fig. S4A) and ER labeling in the cells (Supplementary Fig. S4B). Immunofluorescent labeling in the roots of the *SYT1* null mutant, *syt1-2*, with SYT1-specific antibody undergoing the same procedure showed no fluorescent signals (Supplementary Fig. S4C). Western blot using VAP27-1-specific antibody showed a single band of the expected molecular weight in wild-type Arabidopsis and the *SYT1* null mutant. The intensity of the band was reduced in the *VAP27-1* RNAi knockdown line (Supplementary Fig. S4D). These data indicated that the aforementioned antibodies were specific.

Ultrastructural immunogold labeling for SYT1 (15 nm gold particles) and VAP27-1 (6 nm gold particles) further confirmed that these two proteins were localized on distinct domains of the ER–PM contact sites (Fig. 3B). Gold particles located along the PM were counted and the result showed that 61.82% of the labeled regions were VAP27-1 single positive, 28.22% of the labeled regions were SYT1 single positive, and 9.96% of the labeled regions were VAP27-1/SYT1 double positive. Statistical analyses using the χ^2^ test showed that SYT1- and VAP27-1-positive gold particles tended to localize exclusively on distinct domains. SYT1 showed no preference to cluster with SYT1 itself nor with VAP27-1, whereas VAP27-1 tended to cluster with VAP27-1 itself (Table 1). Based on their respective properties, the two distinct ER–PM contact sites were named as SYT1-enriched ER–PM contact sites (S-EPCSs) and VAP27-1-enriched ER–PM contact sites (V-EPCSs).

**Table 1.** Statistical analysis for clustering of gold particles Gold particles of 15 nm (SYT1) and 6 nm (VAP27-1) are counted along the PM. A 100 × 50 nm area with positive signal(s) is defined as one labeled ER–PM contact site. The contact sites are designated as SYT1 positive (SYT1+), VAP27-1 positive (VAP27+), and SYT1/VAP27-1 double positive (SYT1+VAP27+).

	ER–PM contact sites labeled by gold particles				
	SYT1+	SYT1+VAP27+	VAP27+	Total	
No. of contact sites	68	24	149	241	
	**χ^2^ test for clustering of gold particles**				***P*-value**
No. of SYT1 particles	161 (156.70)	51 (55.20)	–	212	0.5
No. of VAP27-1 particles	–	77 (124.86)	823 (775.14)	900	3.93E-06

χ^2^ tests show that SYT1 particles are randomly distributed on the SYT1+ and SYT1+VAP27+ contact sites, but VAP27-1 particles tend to cluster together on VAP27+ contact sites.

The values in parentheses indicate expected values of random distribution.

### SYT1 stabilizes the V-EPCSs by maintaining the polygonal ER network

Our results showed that SYT1 and VAP27-1 were not co-localized on the ER–PM contact sites. Nevertheless, these two contact sites were closely located; therefore, it was interesting to investigate their anchoring mechanisms. A previous study has shown that point mutations on the MSP domain of VAP27-1 (VAP27-1-T59/60A) reduce the efficiency of PM tethering (Wang *et al.*, 2014). To investigate the localization patterns of the VAP27-1 mutant and SYT1, VAP27-1-T59/60A–YFP was co-expressed with SYT1–GFP in tobacco leaves. Time-lapse imaging showed that the VAP27-1-T59/60A mutant was distributed on the ER network and unable to form a stable EPCS (Supplementary Fig. S5; Supplementary Movie S3). Still, the VAP27-1-T59/60A-labeled ER was connected to the stable S-EPCSs (Supplementary Fig. S5; Supplementary Movie S3), indicating that S-EPCSs might restrain the mobility of the ER network and VAP27-1. However, the expression of VAP27-1 mutant protein did not affect the stability of S-EPCSs.

**Fig. 4.**
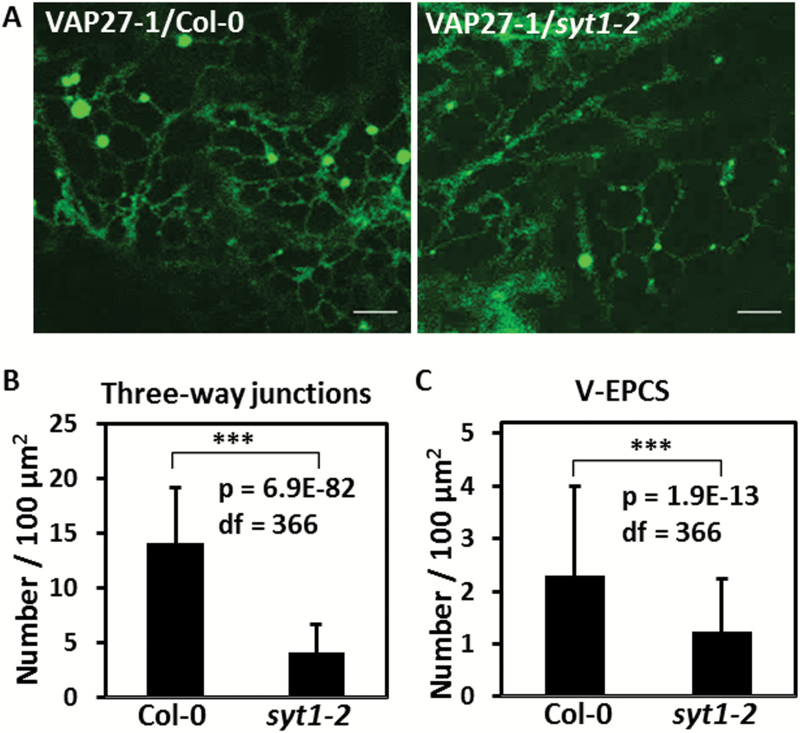
SYT1 is essential for maintaining the polygonal network of the ER. (A) The stable VAP27 puncta are still observed in the leaf cells of VAP27–YFP transgenic Arabidopsis in the *syt1-2* background, but the ER network in the *syt1-2* background is less connected compared with that in the Col-0 background. (B) Comparing the number of the three-way junctions of the ER in the leaf cells of *VAP27-YFP/*Col-0 and *VAP27-YFP/syt1-2* transgenic Arabidopsis shows that the junctions are significantly reduced in the *syt1-2* background (*t*-test, ***P-value <0.001). (C) The number of the VECSs is reduced by 46.61% by the loss of *SYT1* (*t*-test, **P-value <0.001). The three-way junctions and the V-EPCSs were counted per 100 µm^2^ in the first leaf cells of the transgenic plants from the confocal images. In total, 368 of the areas from 64 cells were counted. Scale bars=5 µm. Error bars=SD. (This figure is available in colour at *JXB* online.)

To examine if SYT1 is required for the formation of V-EPCSs, VAP27-1–YFP was stably transformed into Arabidopsis Col-0 and the *SYT1* null mutant, *syt1-2*, using floral dipping. The result showed that V-EPCSs still existed in the leaf epidermal cells of *syt1-2*; however, the ER networks in *syt1-2* were not well connected and the behavior of the V-EPCSs had changed compared with that in the Col-0 background (Fig. 4A). Quantitation of the polygonal network of the ER tubules showed that the number of the three-way junctions was reduced by 70.57% in the leaf cells of *syt1-2* compared with that in Col-0 (Fig. 4B). This result indicated that the cortical ER in *syt1-2* cells was less reticulated. The stable V-EPCSs labeled by VAP27-1–YFP were still recorded in *syt1-2* but showed a 46.61% of reduction in number (Fig. 4C). This result suggested that the establishment and the stability of the V-EPCSs were regulated by SYT1. Fewer EPCSs could be formed in the absence of SYT1, resulting in a reduced number of three-way junctions.

**Fig. 5.**
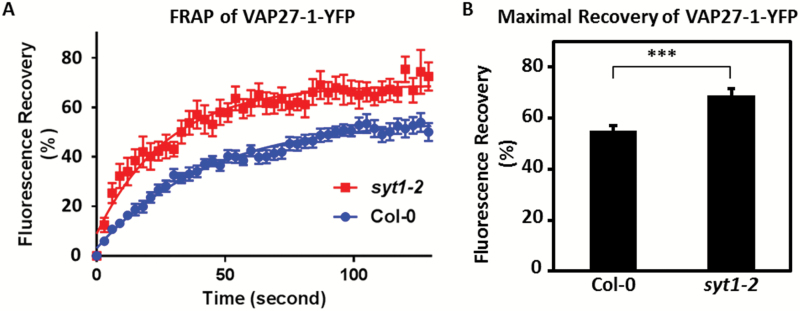
The motility of VAP27-1 at the ER–PM contact sites is restrained by SYT1. (A) FRAP of VAP27-1–YFP at the VECSs in the leaf cells of *VAP27-1-YFP/*Col-0 and *VAP27-1-YFP/syt1-2* transgenic Arabidopsis shows that the maximal recovery of VAP27-1–YFP in the *syt1-2* background is higher than that in the Col-0 background. Note that the error bars are wider in the *syt1-2* background, indicating that the recovery of VAP27 is more varied. Error bars=SE. (B) The diagram shows that the motile fraction of VAP27 is enhanced in the absence of SYT1. The three asterisks indicate a significant difference between the two groups according to the extra-sum-of-squares *F*-test of the best-fit values (*P*-value <0.0001). Thirteen VECSs of each line were analyzed. Error bars=95% confidence intervals. (This figure is available in colour at *JXB* online.)

**Fig. 6.**
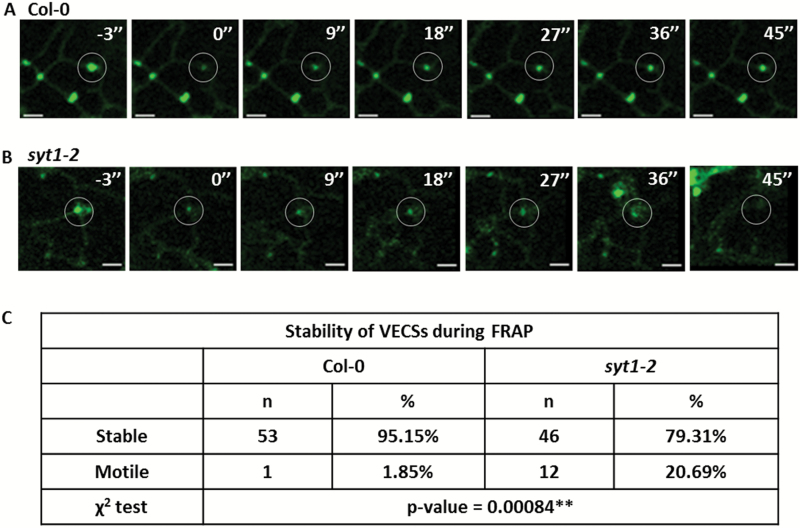
The VAP27-enriched ER–PM contact sites are unstable in the absence of SYT1. (A) Time-lapse images show that the fluorescence of VAP27–YFP is able to recover at the same spot in the Col-0 background. 0'' indicates the first captured image after photobleaching. Scale bars=2 µm. (B) Time-lapse images show one example that a VAP27–YFP punctum moves away while recording the movies in the *syt1-2* background. (C) χ^2^ tests show that there are more unsteady VAP27 puncta in the *syt1-2* null background. (This figure is available in colour at *JXB* online.)

To examine further if the dynamics of VAP27-1 on the V-EPCSs was altered in the absence of SYT1, FRAP of VAP27-1–YFP was performed in the leaves of *VAP27-1-YFP*/Col-0 and *VAP27-1-YFP*/*syt1-2* transgenic Arabidopsis (Fig. 5A). The results showed that the maximal recovery of VAP27-1-YFP in *syt1-2* (69.08 ± 2.48%) was higher than that in Col-0 (55.20 ± 1.73%, *P*-value <0.0001) (Fig. 5B), indicating increased mobility of VAP27-1 in *syt1-2*. In addition, 20.69% (12 out of 58) of the V-EPCSs in *syt1-2* were unstable and motile during FRAP analysis compared with only 1.85% (one out of 54) in the Col-0 background (Fig. 6; Supplementary Movie S4), showing that the V-EPCSs were unstable in the *SYT1* null mutant. In summary, the above data demonstrate that SYT1 is essential for maintaining the polygonal network of cortical ER and the stability/dynamics of VAP27-1 on the ER–PM contact sites.

## Discussion

### Subcellular localization of SYT1

Early studies suggested that SYT1 is a PM protein that can also be found at endosomes (Schapire *et al.*, 2008; Yamazaki *et al.*, 2008; Lewis and Lazarowitz, 2010). However, two recent studies have shown that Arabidopsis SYT1 is an ER integral membrane protein localized on the ER–PM junctions (Levy *et al.*, 2015; Perez-Sancho *et al.*, 2015). The subcellular localization of Arabidopsis SYT1 is convoluted owing to (i) the resemblance of the protein to human SYT1 and E-SYT1; (ii) the phospholipid binding property of the C2 domains and the characteristics of the TM; and (iii) the close proximity between the PM and the cortical ER in plant cells. Arabidopsis SYT1 was deemed functionally related to human SYT1 because both Arabidopsis SYT1 and human SYT1 possess a single N-terminal TM domain and two tandem C2 domains at the C-terminus. Human E-SYTs, different from Arabidopsis SYT1 and human SYT1, have a long N-terminal TM domain that forms a hairpin-like structure, and 3–5 C2 domains at the C-termini. However, human E-SYTs share with Arabidopsis SYT1 a conserved SMP domain located between their TM and the C2 domains (Kopec *et al.*, 2010; Levy *et al.*, 2015). This study demonstrates that Arabidopsis SYT1 is an ER-anchored protein localized on the ER–PM contact sites. Whole-mount fluorescent immunocytochemistry and cryo-immunogold electron microscopy in Arabidopsis root tips further confirm the ER–PM localization of AtSYT1. Moreover, no SYT1-positive signal was found on the Golgi apparatus and the BFA compartments, showing that Arabidopsis SYT1 is not transported to the Golgi apparatus. No evidence has suggested that membrane fusion occurs on the ER–PM contact sites in animal cells, even though hemifusion on the ER–chloroplast contact sites has been suggested (Mehrshahi *et al.*, 2013; Prinz, 2014). Therefore, excluding the possibility of transient translocation of Arabidopsis SYT1 from the ER to the PM, Arabidopsis SYT1 is an ER integral membrane protein localized on the ER–PM contact sites.

Mammalian SYTs and E-SYTs are both highly expressed in neurons; however, the ability of SYTs to translocate through the exocytosis pathway and trigger the membrane fusion makes it functionally different from the ER-retaining E-SYTs. Human E-SYT2 has three C2 domains (C2A–C2B–C2C), and the C2C domain of E-SYT2 is indispensable for the protein’s cortical localization and binding to the PM (Giordano *et al.*, 2013). Arabidopsis SYT1, in comparison, contains only two C2 domains (C2A–C2B) but is still localized on the cortical ER and tethers to the PM. The SMP domain of Arabidopsis SYT1 has also been shown to be critical for the puncta localization (Yamazaki *et al.*, 2010; Perez-Sancho *et al.*, 2015); however, the deletion of the SMP domain in human E-SYT2 has no apparent effect on its localization (Giordano *et al.*, 2013). It seems that Arabidopsis SYTs and mammalian E-SYTs have evolved different sorting and anchoring mechanisms.

### Tethering of SYT1 and VAP27-1 on ER–PM contact sites

The relationships between Arabidopsis SYT1 and the ER–PM-tethering proteins VAP27-1 and NET3C have been addressed in this study. We have confirmed that Arabidopsis SYT1 does not co-localize with VAP27-1 and NET3C on the ER–PM contact sites. The localization patterns of these proteins indicate that there are different types of ER–PM contact sites. These contact sites may have different functions, but their functions are inter-related. From our observation, the V-EPCSs are always associated with the S-EPCSs, but half of the S-EPCSs are not attached to the V-EPCSs, suggesting that the V-EPCSs may be dependent on the S-EPCSs.

**Fig. 7.**
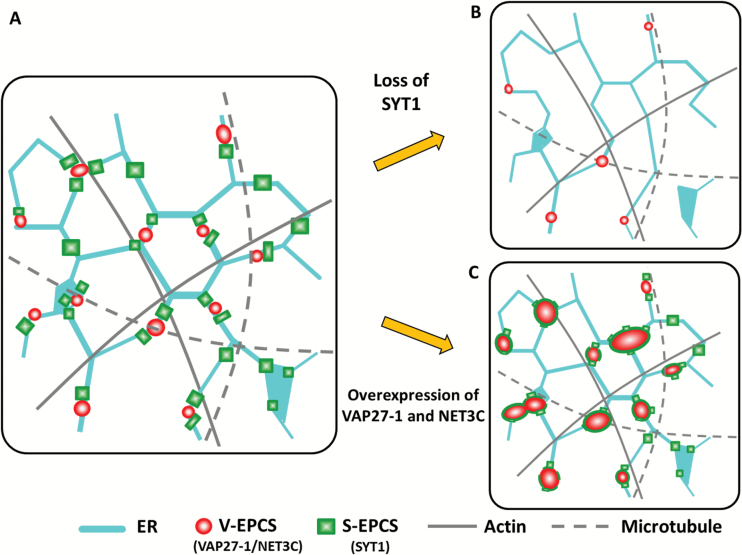
Schematic of SYT1, VAP27-1, NET3C, and cytoskeletons on the cortical ER. (A) VAP27-1 and NET3C are co-localized on the V-EPCSs, which are surrounded by the S-EPCSs. SYT1 maintains the polygonal network of cortical ER and the stability of V-EPCSs by tethering the ER to the PM. S-EPCSs are often arranged along the actin filament but tend to be excluded by the microtubules. ER-resident VAP27-1 interacts with NET3C and microtubules, whereas NET3C interacts with VAP27-1, ER, and PM membranes, and actin on the V-EPCSs. Therefore, V-EPCSs are stabilized by microtubules, NET3C, and S-EPCSs on the cortical ER. (B) The absence of SYT1 protein results in a less stable and reticulated ER network, fewer V-EPCSs, and less stability of VAP27-1 on the V-EPCSs. The reduced stability of the ER network and the EPCSs may give rise to the stress-sensitive phenotypes in the *SYT1* null mutant. (C) Overexpression of VAP27-1 and/or NET3C results in the enlargement of the V-EPCSs, which are encircled by the S-EPCSs. In this model, Arabidopsis ER–PM contact sites can be characterized by a central VAP27-1/NET3C core (V-EPCS) and the SYT1 periphery (S-EPCSs). SYT1 may play roles in creating and maintaining the boundaries of the ER–PM contact sites. (This figure is available in colour at *JXB* online.)

Our results show that the tethering of VAP27-1 to the PM does not require SYT1 because the two proteins are not co-localized and the V-EPCSs can still be found in the *SYT1* null mutant. However, the ER tubules are more dynamic, the V-EPCSs are less stable, and the turnover of VAP27-1 increases in the *SYT1* null mutant. The ER network is less connected and the average number of three-way junctions is decreased in the absence of SYT1 (Fig. 7), although cells with well-reticulated ER and those with extremely motile ER strands can also be observed in the same leaf. Based on the above observations, it can be inferred that Arabidopsis SYT1 plays important roles in stabilizing the ER network or supporting the compartmentation of the cell cortex.

### Patterns of ER–PM contact sites and vesicle trafficking

Previous studies have indicated that the ER–PM contact sites in nerve cells also display different forms and shapes: the ER–PM junctions show discrete punctate patterns at the synapses, whereas the width of an ER–PM contact site can extend up to 2–4 µm in other regions of the neurons (Rosenbluth, 1962; Hayashi *et al.*, 2008; Stefan *et al.*, 2013). Moreover, it has been shown that the sizes of the S-EPCSs in Arabidopsis leaf cells are increased by mechanical stress (Perez-Sancho *et al.*, 2015). The expression of SYT1–GFP driven by the native *SYT1* promoter in tobacco leaves also shows various patterns and sizes of the S-EPCSs on the cell cortex. The immunofluorescent labeling of SYT1 in the root cells also shows various sizes of the S-EPCS in our observation. The patterns may result from different expression levels of SYT1 and certain regulatory events in the cells.

A previous study has shown that the formation of PM-derived endosomes is inhibited by the expression of truncated SYT1 lacking the C2B domain in *N. benthamiana* leaf cells (Lewis and Lazarowitz, 2010). Arabidopsis SYT1 has also been shown to participate in plasma membrane resealing (Yamazaki *et al.*, 2008), which is mediated by Ca^2+^-dependent exocytosis in animal cells (McNeil and Kirchhausen, 2005). In addition, Arabidopsis SYT1 interacts with the PM syntaxin penetration 1 (PEN1) and negatively regulates the immune secretory pathways in response to powdery mildew fungi, probably via modulating the ER–PM contact sites (Kim *et al.*, 2016).

This study has demonstrated that the fluorescence recovery of VAP27-1–YFP after photobleaching on the ER–PM contact sites is enhanced in the *SYT1* null mutant. Because the enhanced mobility of VAP27-1 on the V-EPCSs couples with the enhanced ER remodeling in the *SYT1* mutant, it can be inferred that Arabidopsis SYT1 restrains the ER movement by ER–PM tethering and then stabilizes the V-EPCSs without a direct interaction with VAP27-1. Taken together, these data suggest that Arabidopsis SYT1 plays a role in maintaining the ER stability and in regulating vesicle trafficking by controlling the extent of ER–PM contact sites. Recently, different types of proteins localized on the ER–PM contact sites have also been discovered in yeast and human (Gatta *et al.*, 2015; Henne *et al.*, 2015). Further studies on ER–PM anchor proteins in plants will help to reveal the complexity of ER–PM interactions.

## Supplementary data

Supplementary data are available at *JXB* online.

Figure S1. SYT1 and VAP27-1 are not localized to the Golgi apparatus and clathrin-coated vesicles.

Figure S2. NET3C is co-localized with VAP27-1 on the V-EPCSs.

Figure S3. Spatial relationships between SYT1, VAP27-1, and the cytoskeletons.

Figure S4. SYT1 and VAP27 antibodies are specific.

Figure S5. VAP27-1-T59/60A does not interrupt the formation of S-EPCSs.

Movie S1. Co-expression of SYT1–GFP and RFP–HDEL.

Movie S2. Co-expression of SYT1–GFP and VAP27-1–YFP.

Movie S3. Co-expression of SYT1–GFP and VAP27 mutant–YFP.

Movie S4. The V-EPCSs are more motile in the Arabidopsis *SYT1* null mutant.

Supplementary Data
