# Supplementary material for: Arabidopsis SYT1 maintains stability of cortical endoplasmic reticulum networks and VAP27-1-enriched endoplasmic reticulum–plasma membrane contact sites
Source: J Exp Bot. 2016 Oct 17;67(21):6161–71. doi: 10.1093/jxb/erw381 (PMC5100027; doi:10.1093/jxb/erw381)
Supplement: Supplementary Data [file supp_67_21_6161__index.html]

Arabidopsis SYT1 maintains stability of cortical endoplasmic reticulum networks and VAP27-1-enriched endoplasmic reticulum–plasma membrane contact sites — Arabidopsis SYT1 maintains stability of cortical endoplasmic reticulum networks and VAP27-1-enriched endoplasmic reticulum–plasma membrane contact sites — Supplementary Data 

# Arabidopsis SYT1 maintains stability of cortical endoplasmic reticulum networks and VAP27-1-enriched endoplasmic reticulum–plasma membrane contact sites

## Supplementary Data

Data files

- supplementary\_Figures\_S1\_S5.pdf - Supplementary Data
- supplementary\_Movie\_S1.mov - Supplementary Data
- supplementary\_Movie\_S2.mov - Supplementary Data
- supplementary\_Movie\_S3.mov - Supplementary Data
- supplementary\_Movie\_S4.mov - Supplementary Data
