# Supplementary material for: Arabidopsis SYT1 maintains stability of cortical endoplasmic reticulum networks and VAP27-1-enriched endoplasmic reticulum–plasma membrane contact sites
Source: J Exp Bot. 2016 Oct 17;67(21):6161–71. doi: 10.1093/jxb/erw381 (PMC5100027; doi:10.1093/jxb/erw381)
Supplement: Supplementary Data [file supp_erw381_supplementary_Figures_S1_S5.pdf]

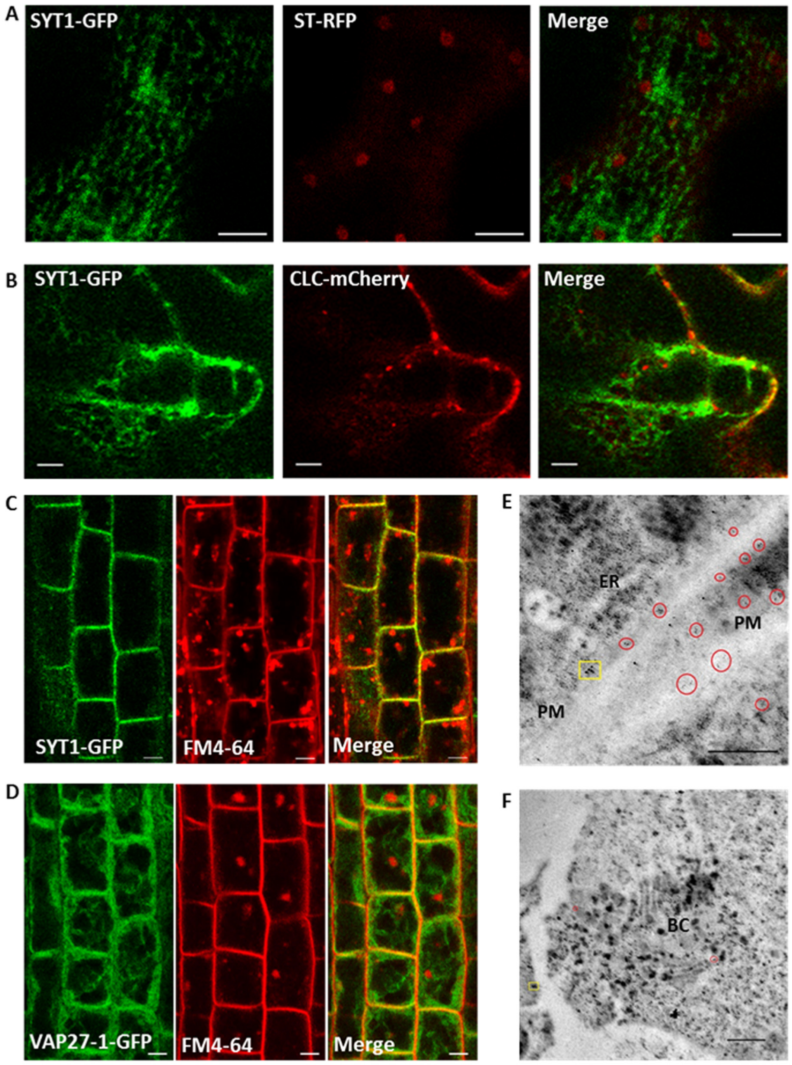

Supplementary Fig. S1. SYT1 and VAP27-1 are not localized to the Golgi apparatus and clathrin-coated vesicles. (A) Co-expression of SYT1-GFP and ST-RFP in tobacco leaves shows that SYT1 is not translocated to the Golgi apparatus. Scale bars = 5 µm. (B) SYT1-GFP is not incorporated into the CLC-labeled early endosomes. (C) FM4-64 staining of the roots of SYT1-GFP transgenic Arabidopsis followed by BFA treatment (35.6 µM) for 60 min shows that SYT1 is not localized to the BFA compartments. Scale bars = 5 µm. (D) VAP27-1-GFP is not co-localized with the BFA compartments in the root cells of VAP27-1-GFP transgenic Arabidopsis. Scale bars = 5 µm. (E) Double immunogold labeling of the BFA-treated roots of wild type Arabidopsis shows that both SYT1 and VAP27-1 are still localized on the ER-PM contact sites. Scale bars = 500nm. (F) The electron microscopic image shows an aggregate of Golgi stacks and vesicles (BFA compartment, B) in the BFA-treated root cells. No gold particles are observed in the BFA compartment by double immunogold labeling. 15-nm gold particles (SYT1) are indicated by a yellow rectangle and 6-nm gold particles (VAP27-1) are indicated by red circles. Scale bars = 500nm.

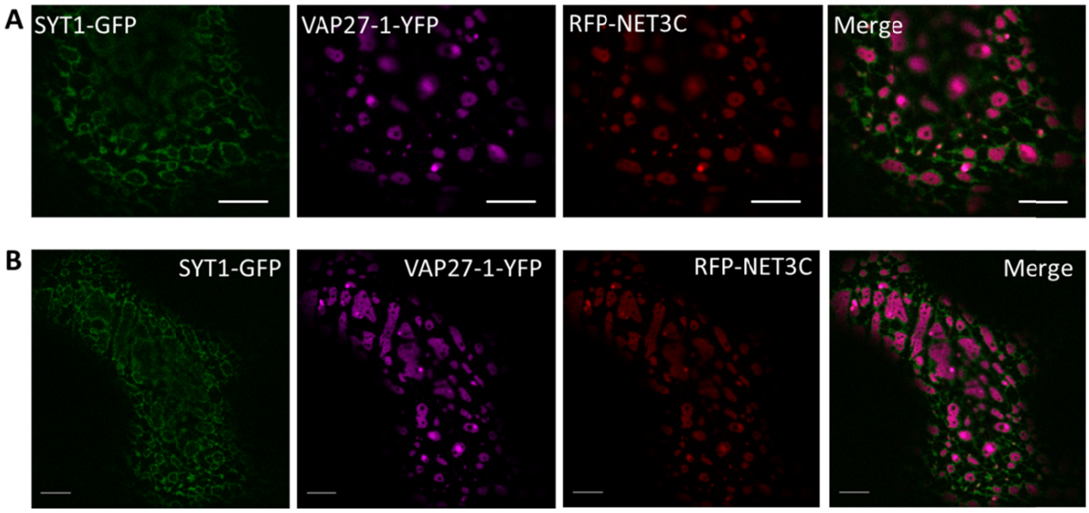

Supplemental Fig. S2. NET3C are co-localized with VAP27-1 on the V-EPCSs. (A) Co-expression of SYT1-GFP, VAP27-1-YFP, and RFP-NET3C shows that SYT1 is excluded from the VAP27-1/NET3C-localized ER-PM contact sites. (B) The images show another region of the cell in (A) with the wide-spreading V-EPCSs. Scale bars = 5 µm.

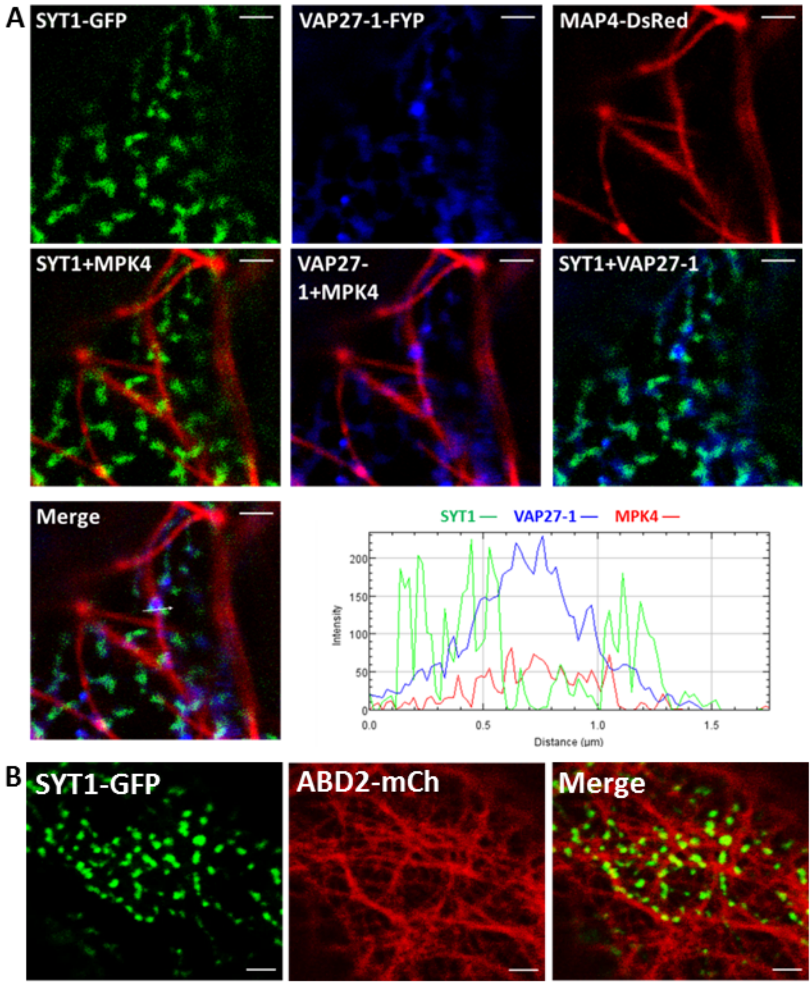

Supplemental Fig. S3. Spatial relationships between SYT1, VAP27-1, and the cytoskeletons. (A) Co-expression of SYT1-GFP, VAP27-1-YFP, and MPK4-DsRed in *N. benthamiana* leaves shows that the localizations of SYT1 and VAP27-1 are associated with the microtubules. The intensity profiles of one VAP27 punctum, two SYT1 puncta, and one microtubule show that VAP27-1 overlaps with the microtubule and is sandwiched by the SYT1 puncta. (B) The SYT1 puncta are often overlapped with the ABD2-labeled actin filaments. Scale bars = 2 µm.

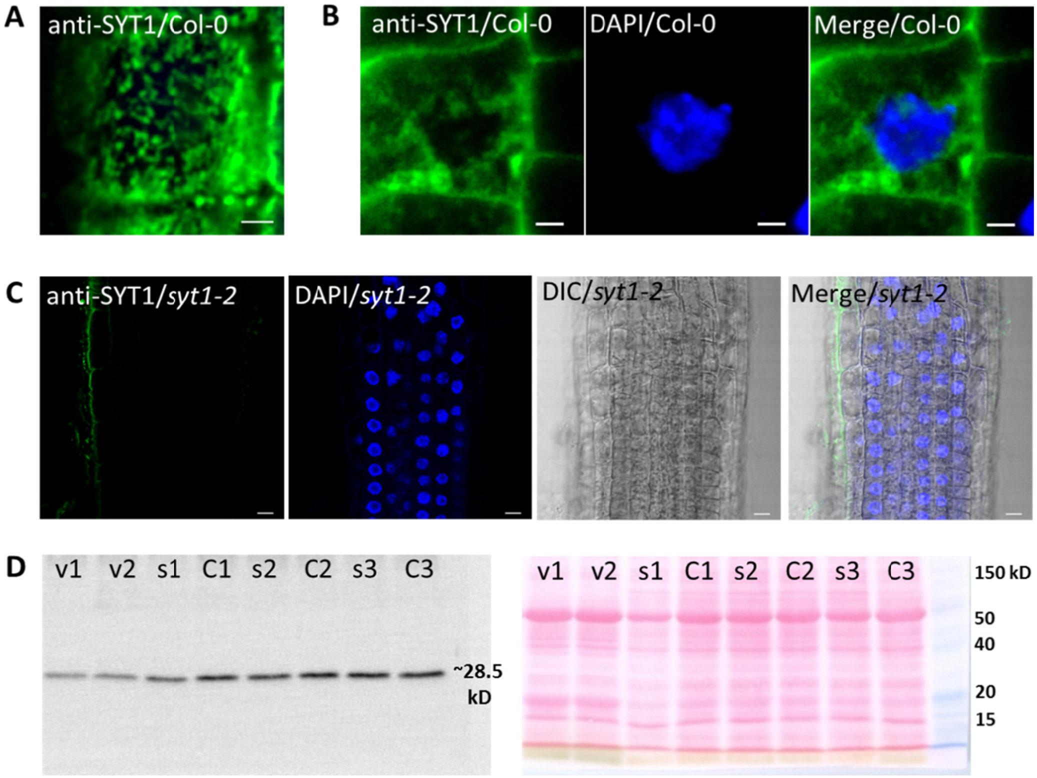

Supplemental Fig. S4. SYT1 and VAP27 antibodies are specific. (A) Immunofluorescent labeling of SYT1 in wild type Col-0 shows that SYT1 forms punctate structures on the cell cortex in the root cells. Scale bar = 2 µm. (B) SYT1 is localized on the ER through the cytoplasm and around the nucleus. Scale bars = 2 µm. (C) Immunofluorescent labeling using SYT1 antibody in *syt1-2* null mutant shows no positive signal. Scale bars = 10 µm. (D) Western blot of proteins from 5-day-old seedlings of Col-0 (C1 to C3), *syt1-2* (s1 to s3), and VAP27-1 RNAi mutant (v1 and v2) using VAP27-1 antibody shows one single band in each line (Left). The blotted proteins on the PVDF membrane are stained by Ponceau S (Right).

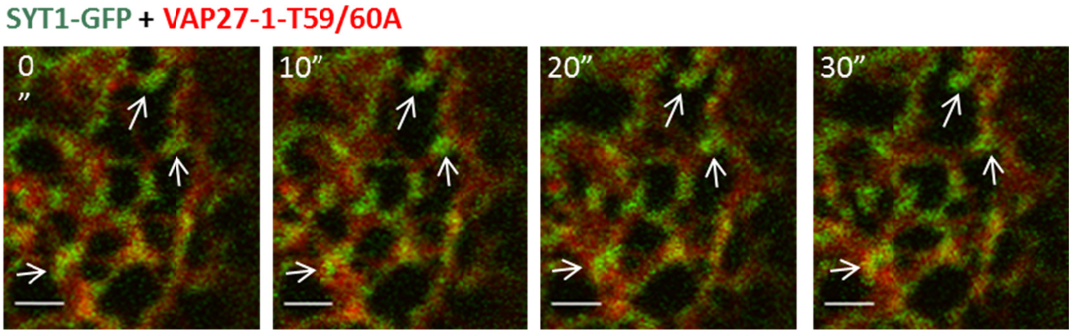

Supplemental Fig. S5. VAP27-1-T59/60A does not interrupt the formation of S-EPCSs. The time-lapse imaging shows that VAP27-T59/60A (red) proteins are unable to stably anchor on the ER-PM contact sites. However, SYT1 (green) is unaffected by the VAP27-1 mutant and still able to form stable S-EPCEs (arrows). Scale bars = 2 µm.
